# 

**Published:** 1867-06

**Authors:** 


### Books and Pamphlets Received.

Circular No. 5: War Department, Surgeon General's Office, May 4th, 1867.

Report one pidemic cholera, in the army of the United States, during the year 1866.

Notes on the origin, prevention and treatment of Asiatic cholera. By John C. Peters, M. D. Second edition, with an appendix. New York: D. Van Nostrand, 192 Broadway, 1867.

Ununited fracture successfully treated, with remarks on the operation. By Henry J. Biglow, M. D., Professor of Surgery in the Medical College of Harvard University. With abstracts from Dr. Biglow's Clinical Lectures on the subject and cases.

Women as Physicians.

Introductory Address on the Commencement of the Session of the Medical Department of the Willamette University, for the year 1867. By A. Sharples, A. B., M. D., Professor of Anatomy, Salem, Oregon.

Sixth Annual Report of the Board of Managers of the Woman's Hospital of Philadelphia. 1867.

Eighteenth Annual Announcement of the Woman's Medical College of Pennsylvania, 1867-68.

Lecture on the physiology of the heart and its connections with the brain, delivered at a meeting at the Sorbonne, the 27th March, 1865. Translated by J. S. Moore, M. D., Savannah, Ga.

